# Linking bacterial community composition to soil salinity along environmental gradients

**DOI:** 10.1038/s41396-018-0313-8

**Published:** 2018-11-16

**Authors:** Kristin M. Rath, Noah Fierer, Daniel V. Murphy, Johannes Rousk

**Affiliations:** 1 Department of Biology, Section of Microbial Ecology, Lund University, Lund, Sweden; 2 Centre for Environmental and Climate Research (CEC), Lund University, Lund, Sweden; 3 Cooperative Institute for Research in Environmental Sciences and Department of Ecology and Evolutionary Biology, University of Colorado, Boulder, CO USA; 4 SoilsWest, UWA School of Agriculture and Environment, The University of Western Australia, Crawley, WA Australia

**Keywords:** Microbial ecology, Biodiversity

## Abstract

Salinization is recognized as a threat to soil fertility worldwide. A challenge in understanding the effects of salinity on soil microbial communities is the fact that it can be difficult to disentangle the effects of salinity from those of other variables that may co-vary with salinity. Here we use a trait-based approach to identify direct effects of salinity on soil bacterial communities across two salinity gradients. Through dose–response relationships between salinity and bacterial growth, we quantified distributions of the trait salt tolerance within the communities. Community salt tolerance was closely correlated with soil salinity, indicating a strong filtering effect of salinity on the bacterial communities. Accompanying the increases in salt tolerance were consistent shifts in bacterial community composition. We identified specific bacterial taxa that increased in relative abundances with community salt tolerance, which could be used as bioindicators for high community salt tolerance. A strong filtering effect was also observed for pH across the gradients, with pH tolerance of bacterial communities correlated to soil pH. We propose phenotypic trait distributions aggregated at the community level as a useful approach to study the role of environmental factors as filters of microbial community composition.

## Introduction

One of the major challenges in the field of microbial ecology is to move beyond descriptive reports of patterns in community composition and find a basis for predicting shifts in bacterial communities in response to environmental changes [1]. Unfortunately, our understanding of the underlying dynamics that structure bacterial communities remains limited. Differences in community composition have primarily been linked to edaphic factors based on correlative relationships [2]. In a global survey of bacterial communities from both terrestrial and aquatic environments, salinity emerged as the dominant factor linked to bacterial community composition [3] and has also been reported to be the factor most strongly correlated with community composition in aquatic systems [4, 5]. In soil, a large proportion of the variance in overall soil bacterial community composition has been shown to be associated with differences in soil pH [6, 7]. Despite the widespread occurrences of saline soils, much of our understanding of the role of salinity as a driver of bacterial communities has been derived from aquatic systems, with far fewer reports of salinity responses along soil salinity gradients.

Globally, it is estimated that around 900 million hectares of soil are affected to some degree by high ion concentrations [8]. Saline soils, commonly defined as soils with an electrical conductivity (EC) in a saturated soil extract of >4 dS m^−1^ [9], cover a large area in arid and semi-arid regions of the globe. ECs in saline soils can reach >200 dS m^−^^1^ [10], matching the EC of the most saline bodies of water. Considering that soil salinity can span several orders of magnitude (from <0.1 dS m^−^^1^ in non-saline soils to >100 dS m^−^^1^ in highly saline soil), it should be expected that salinity has a similarly strong effect on bacterial community composition in soil as has been observed in aquatic systems and that soil salinity could be another strong driver of microbial community composition in addition to soil pH. Indeed, studies on the composition of bacterial communities of saline soils found shifts in community composition associated with salinity, as well as a decline in phylogenetic diversity as salinity increased [11–14].

When identifying environmental drivers of bacterial community composition based on correlative relationships, the influence of confounding factors is difficult to tease apart from the direct effect of the environmental factor of interest. For instance, soil salinity is frequently correlated with reduced organic matter (OM) input [15, 16] and differences in soil pH [17]. To identify direct effects of environmental factors on the community, trait-based approaches have been proposed [18, 19]. However, information on phenotypic traits at the resolution of single bacterial taxa is often unavailable [20], especially in soil where most taxa remain undescribed [21]. Measuring traits aggregated at the community level can be used as an alternative to measure the distribution of traits within a community [18, 22]. This approach has been used to study microbial trait distributions including those of pH tolerance [23, 24], salt tolerance [25, 26], and heavy metal tolerance [27] among others. Tolerance to heavy metal pollution measured at the level of communities has been linked to shifts in community composition [28, 29], which makes it possible to infer a causal connection between shifts in the abundance of microbial taxa and changes in community tolerance. Shifts in environmental community tolerance indicate that the environmental factor in question effectively filtered the bacterial community. Changes in bacterial community composition that correlate with increases in community salt tolerance are thus far stronger indicators of a causal link between soil salinity and community composition than correlations between salinity and bacterial community compositions alone.

In this study, we tested the associations between community composition and the distribution of the trait salt tolerance along gradients of soil salinity. We used two salinity gradients, of which one was confounded by a pH gradient. We also established pH optima for the communities to investigate whether soil pH had selected for a shift in the trait distribution of pH tolerance and thus also investigated if pH had filtered bacterial community structure. We hypothesized (i) that the local environment would have selected for a community with matched trait distributions for salt tolerance and pH tolerance, (ii) that differences in salt tolerance and pH tolerance would be reflected in differences in the community composition, and (iii) that changes in bacterial community composition observed with increasing salt tolerance of the community would be consistent across both gradients.

## Material and methods

### Sites

Bulk soil samples were collected along two salinity gradients located along Lake O’Connor in Western Australia. Each gradient was sampled in three transects of eight sampling points. At each sampling point, 6 soil cores (5 cm diameter, 5 cm depth) within a 2 m radius were combined into a composite sample and stored in polyethylene Ziploc bags. This resulted in a total of 24 samples collected for each gradient (Fig. S1). Along the gradients, vegetation changed with distance from the lakeshore. On the northern shore gradient (henceforth the agricultural [AG] gradient; 32°28’ S, 119°13’ E), the first sampling point of each transect was located at the edge of the salt lake. The vegetation at sampling point 1 consisted of small halophilic shrubs with little ground cover. At sampling points 2–4, halophilic species were gradually replaced by grasses and small trees (*Allocasuarina* sp. and *Eucalytpus* sp.), while ground cover increased with distance from the lakeshore. Sampling points 5–8 were located in agricultural land used for wheat production. The distance covered by each transect of the AG gradient was ca. 200 m. The distance between transect A and B was ca. 130 m and between B and C ca. 560 m.

On the southern shore gradient (henceforth the natural vegetation [NV] gradient; 32°30’ S, 119°13’ E), sampling point 1 was also located at the lakeshore, with a vegetation consisting of mostly halophilic shrubs. At sampling point 2, vegetation consisted of grasses and *Allocasuarina* sp. From samplings points 3–6, *Eucalyptus* sp. became more common, with increasing ground cover. Sampling points 7–8 were covered with non-saline *Eucalyptus* woodland. Each transect of the NV gradient covered a distance of ca. 300 m from the lakeshore to the last sampling point. The distance between transects was ca. 300 m. The AG and NV gradients were located about 2.5 km apart.

Soil EC and pH were determined in a 1:5 soil: water mixture. OM contents were obtained as loss-on-ignition (600 °C, 12 h). The amount of water-soluble cations (Ca^2+^, K^+^, Mg^2+^, Na^+^) was analyzed in water extractions from soil with inductively coupled plasma–optical emission spectroscopy (Optima 8300, Perkin Elmer).

### Determination of trait distributions of salt and pH tolerance

Prior to measuring community salt and pH tolerance, soils were incubated with 5 mg g^−^^1^ alfalfa–straw mixture for 3 weeks at room temperature to boost bacterial growth rates. After this pre-incubation period, assays to determine the trait distributions of salt and pH tolerance were performed. Soil suspensions were created by mixing 1 g of soil with 20 ml of water. After homogenization and centrifugation (1000 × *g*), aliquots (1.35 ml) of bacterial suspension were transferred to 2 ml microcentrifugation tubes and were then adjusted to either different electrical conductivities (suspension EC) or different pH values (suspension pH).

To change suspension EC, the soil suspensions were mixed with a range of solutions of different concentrations of NaCl dissolved in water to create a gradient of 8 different salt concentrations (including one control level without salt addition) in a final volume of 1.5 ml. Target concentrations of NaCl additions ranged from 5.5 to 0.007 mol l^−^^1^, with the achieved suspension EC depending on the initial EC of the soil suspension. To adjust suspension pH, suspensions were mixed with 0.15 ml of a citrate–phosphate pH buffer including 11 different levels ranging from pH 3.0 (final concentration 1.1 mM K_2_HPO_4_ and 0.5 mM citric acid) to 8.0 (final concentration 0.25 mM KH_2_PO_4_ and 6.4 mM K_2_HPO_4_) or distilled H_2_O, resulting in a range of 12 different pHs for each sample. The suspension pH was validated with a pH meter. The used buffer concentrations did not affect bacterial Leu incorporation rates within the short time frame studied [30]. Following the adjustment of salinity or pH in the soil suspensions, bacterial growth was measured as the incorporation of ^3^H-labeled leucine into bacterial protein [31, 32]. Briefly, 2 µl of radioactively labeled leucine, ([^3^H]Leu, 185 MBq ml^−^^1^, 2 TBq mmol^−^^1^, Perkin Elmer) were added together with non-labeled leucine to the samples, resulting in a total concentration of 280 nM leucine. After 1 h incubation at room temperature, growth was terminated by the addition of trichloroacetic acid (TCA). After a series of washing steps using TCA and ethanol, the amount of incorporated ^3^H-label was determined through liquid scintillation [32].

### DNA extraction, amplification, and sequencing

DNA was extracted from all soil samples before the addition of plant material used to boost growth rates for tolerance measurements (see above) and from two thirds of samples after they had been incubated with plant material for 3 weeks to check if the incubation period had resulted in community shifts. Subsamples of each soil sample were freeze-dried and ground. DNA was extracted from portions of 250 mg of homogenized ground soil using the MoBio PowerSoil DNA Isolation Kit (Carlsbad, CA, USA) according to the manufacturer’s recommendations. Extracted DNA was amplified using the 16S rRNA gene primer pair 515-F (5′-GTGCCAGCMGCCGCGGTAA-3′) and 806-R (5′-GGACTACHVGGGTWTCTAAT-3′) targeting the V4 region of the 16 S rRNA gene, which included Illumina adapters and unique barcode sequences for each sample. PCR was performed with GoTaq® Hot Start PCR Master Mix (Promega, Madison, WI, USA) in a 25 μl reaction. Thermal cycling consisted of an initial denaturation step at 94 °C for 3 min, followed by 35 cycles of denaturation at 94 °C (45 s), annealing at 50 °C (30 s), extension at 70 °C (90 s), and a final extension at 72 °C for 10 min. The amplified DNA was sequenced using a Illumina MiSeq platform (Illumina, San Diego, CA, USA).

Sequences were processed using the UPARSE pipeline [33] as described in Ramirez et al. [34]. Sequences were quality filtered and clustered de novo into operational taxonomic units (OTUs) at a 97% similarity level. Taxonomic information was assigned to OTUs using the 16S rRNA Greengenes database [35]. To correct for differences in sequencing depth, samples were rarefied to 10,000 reads. Samples with <10,000 reads and OTUs that were observed <10 times across all samples were excluded from downstream analyses. These criteria resulted in the removal of 4 out of the 48 samples and 8928 of the 12,326 OTUs.

### Data analysis

Growth rates in the salt and pH tolerance assays were normalized to growth rates measured at the optimum EC or pH for each sample. In samples in which bacterial growth was inhibited only by increasing salinity, dose–response relationships were established using a logistic model, *Y* = *c*/[1 + *e*^*b*(*x*^^−^^*a*)^], where *Y* is the leucine incorporation rate, *x* is the logarithm of the suspension EC, *a* is the logIC_50_, *c* is the bacterial growth rate in the control without added salt, and *b* is a slope parameter indicating the rate of inhibition. In samples in which growth was inhibited by both increasing and decreasing salinity from its growth optimum, a double-logistic model [23] was used: *Y* = *c*_opt_/(1 + exp[*b*_low-EC_(*x* – *a*_(low-EC)_)]) + *c*_opt_/(1 + exp[*b*_high-EC_(*x* – *a*_high-EC_)]) − *c*_opt_, with *c*_opt_ the growth rate at optimal suspension EC, *b* the slope indicating the rate of decrease toward higher or lower suspension EC, and *a* the logIC_50_ toward higher and lower suspension EC. For comparison of salt tolerance between curves, the logIC_50_ toward higher suspension EC was used. To estimate the pH tolerance of the communities, the same double-logistic model was used, with suspension pH replacing suspension EC as the predictor variable. The suspension pH at which bacterial growth reached its optimum (pH_opt_) in each sample was used as an indicator of the community-level trait distribution of pH tolerance. Kaleidagraph 4.5.0 for Mac (Synergy software) was used to fit the logistic and double-logistic models. Linear regression models were used to test for significant correlations between community-level trait indicators (logIC_50_ and pH_opt_) and soil EC and pH.

The diversity of each sample was determined by calculating the Shannon diversity index. Multivariate statistics were performed in the R environment version 3.3.1 (R [36]) using the ‘vegan’ package [37]. The differences in overall community composition between samples were calculated using the Bray–Curtis dissimilarity index after Hellinger transformation [38]. To highlight the relationship between changes in community tolerance and shifts in community composition, a constrained ordination was performed by distance-based redundancy analysis using *capscale* (vegan) with logIC_50_, pH_opt_, and gradient as constraining variables. In addition, patterns in bacterial community compositions were also visualized by applying an unconstrained ordination method (principal coordinate analysis). The significance of constraining variables was tested with a permutation test (number of permutations = 10,000) using the *anova* function of the vegan package. Correlations between community composition and distributions of salt and pH tolerance were tested using Mantel tests between Bray–Curtis distance matrices of community composition and Euclidean distance matrices of trait distributions. To identify important OTUs correlated with trait distributions, we selected OTUs with a relative abundance of ≥1% in at least one sample. For these OTUs, we calculated Spearman’s rank correlations with both logIC_50_ and pH_opt_. OTUs with a Spearman’s rank correlation coefficient (*ρ*) of ≥0.5 or ≤−0.5 were selected as being positively or negatively correlated with a certain trait. To test for significant correlations between diversity and environmental variables, multiple linear regressions (*α* = 0.05) were performed for each gradient followed by analysis of variance using type II Sums of Squares, with Shannon diversity as the dependent variable and the logarithm of EC and pH as independent variables.

## Results

### Characterization of gradients

The AG gradient encompassed soil ECs ranging from 0.1 to 3 dS m^−^^1^ measured in a 1:5 soil:water mixture, while the soil pH along the gradient was between ca. 5.5 and ca. 7.0 for all but one sample, which had a pH > 8 (Fig. S2). The NV gradient encompassed soil ECs ranging from 0.2 to 9 dS m^−^^1^. Soil pH along the gradient ranged from ca. 4.5 to ca. 8.5. Along the NV gradient, there was a significant negative linear correlation between soil EC and soil pH (*R*^2^ = 0.50, *p* < 0.001), whereas along the AG gradient soil EC and soil pH were not significantly correlated (Fig. S2). OM content along the AG gradient ranged from 2.8 to 4.8% dry weight (dw) and was positively correlated with soil pH (*R*^2^ = 0.26, *p* < 0.01; Fig. S3) but not with soil EC (not shown). Along the NV gradient, OM content ranged from 7.6 to 10.0% dw and was neither correlated with soil EC nor soil pH (Fig. S3). Na^+^ was the predominant cation in sites from both gradients (Fig. S4).

### Community trait distributions

**Fig. 1:**
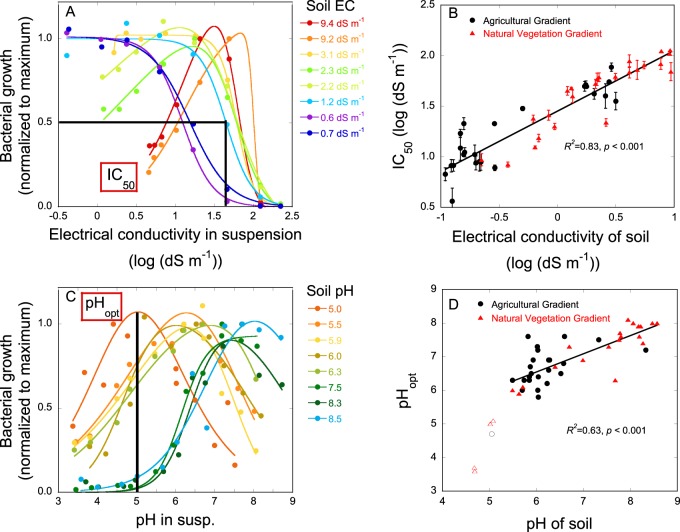
Response curves between bacterial growth and (**a)** suspension electrical conductivity (EC) and (**b)** suspension pH in the community tolerance assays. Depicted are representative examples of response curves of soils of different soil EC and soil pH. Values of soil EC (**a**) and soil pH (**b**) measured in a 1:5 soil:water mixture of the selected soils are given in the figure legends. Bacterial growth was normalized to the maximum growth rate in each sample. The relationships between bacterial growth and EC and pH were described with logistic or double logistic functions. To describe the community-level salt tolerance in a sample, the IC_50_ value (suspension EC at which bacterial growth was inhibited by 50% compared to the maximum) was used. To describe the community-level pH tolerance, the suspension pH at which growth reached its maximum was used (pH_opt_). (**c)** Relationship between soil EC and community-level salt tolerance (indicated by IC_50_) along both salinity gradients (AG and NV gradient). (**d)** Relationship between soil pH and community-level pH tolerance (indicated by pH_opt_). Samples with a pH < 5.5 (open symbols) were excluded from the regression analysis

In samples from sites with lower salinity, bacterial growth was inhibited with increasing salinity (Fig. 1a). The sigmoidal relationship between growth and the logarithm of the suspension EC could be modeled with a logistic function (*R*^2^ from 0.95 to 0.99). In communities from high-salinity samples, bacterial growth was inhibited by both low and high suspension EC (Fig. 1a). For these samples, a double-logistic function could fit the relationships between suspension EC and bacterial growth well (*R*^2^ from 0.91 to 0.99). In the most saline samples, the suspension EC at which communities had their growth optimum was about a factor of 10 higher than that measured in a 1:5 soil:water suspension.

We used the logIC_50_ as an indicator of the community-level distribution of the trait of salt tolerance. There was a strong positive relationship between the logarithm of the soil EC and the logIC_50_ (*R*^2^ = 0.83, *p* < 0.001; Fig. 1c). The difference between soil EC and IC_50_ decreased with increasing salinity. In the most saline samples, the IC_50_ was about 10 times higher than the soil EC, whereas in the least saline samples IC_50_ was 70 times higher than soil EC.

The relationships between bacterial growth and suspension pH could also be modeled with a double-logistic function (*R*^2^ from 0.46 to 0.99, with a mean *R*^2^ = 0.91; Fig. 1b). The indicator used for the distribution of the trait pH tolerance was the pH_opt_ for bacterial growth. Above soil pH 5.5, there was a linear relationship between soil pH and pH_opt_ (*R*^2^ = 0.63, *p* < 0.001; Fig. 1d). For every 1 unit increase in soil pH, the optimum pH increased by ca. 0.5 units. Consequently, around soil pH 5.5, pH_opt_ was around 0.8 units higher than the soil pH, whereas around soil pH 8.5 pH_opt_ was around 0.6 units lower than the soil pH.

### Community composition

In total, 3035 OTUs occurred with a frequency of at least 10 reads in the dataset. In the samples from the AG gradient, we found 2020 different OTUs and in the NV gradient 1897 different OTUs. Of these OTUs, 882 were found in samples from both gradients, whereas the rest were unique to either gradient. In all, 97% of reads belonged to Bacteria, and 3% were assigned to Archaea. The Archaea found along the gradients primarily belonged to two groups, namely, the Thaumarcheota (74% of archaeal reads) and the Halobacteria (20% of archaeal reads). Thaumarcheota were found in samples of all salinities, but were less common in highly saline sites (47% of archaeal reads in samples of salinities >4 dS m^−^^1^), whereas Halobacteria increased in relative abundance with high salinity (35% of archaeal reads in samples of salinities >4 dS m^−^^1^). The phylum Proteobacteria made up 41% of reads, followed by Bacteroidetes (15%), Actinobacteria (14%), Gemmatimonadetes (6%), and Planctomycetes (5%). The most abundant family was the Sphingomonadaceae, a family of Alphaproteobacteria, which accounted for 7% of all reads. Other families that made up >3% of reads were the Chitinophagaceae (Bacteroidetes) and the Xanthomonadaceae (Gammaproteobacteria).

**Fig. 2:**
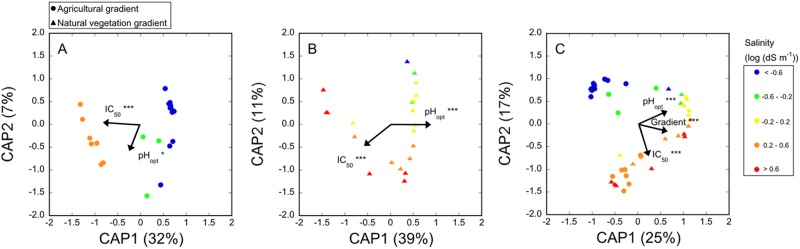
Canonical analysis of principal coordinates (CAPSCALE) derived from Bray–Curtis dissimilarities of the community composition of sampling points based on 16S rRNA gene amplicon sequencing. Panel (**a)** shows samples from the AG gradient, (**b)** shows samples from the NV gradient, and (**c**) shows samples from both gradients. Numbers in parentheses in the axis labels give the percentage of variance accounted for by the principal coordinates. Community salt tolerance (IC_50_), community pH tolerance (pH_opt_), and a dummy variable for gradient were used as constraining variables. The significance of constraining variables was tested with PERMANOVA and is indicated by asterisks next to the variable names.

**Fig. 3:**
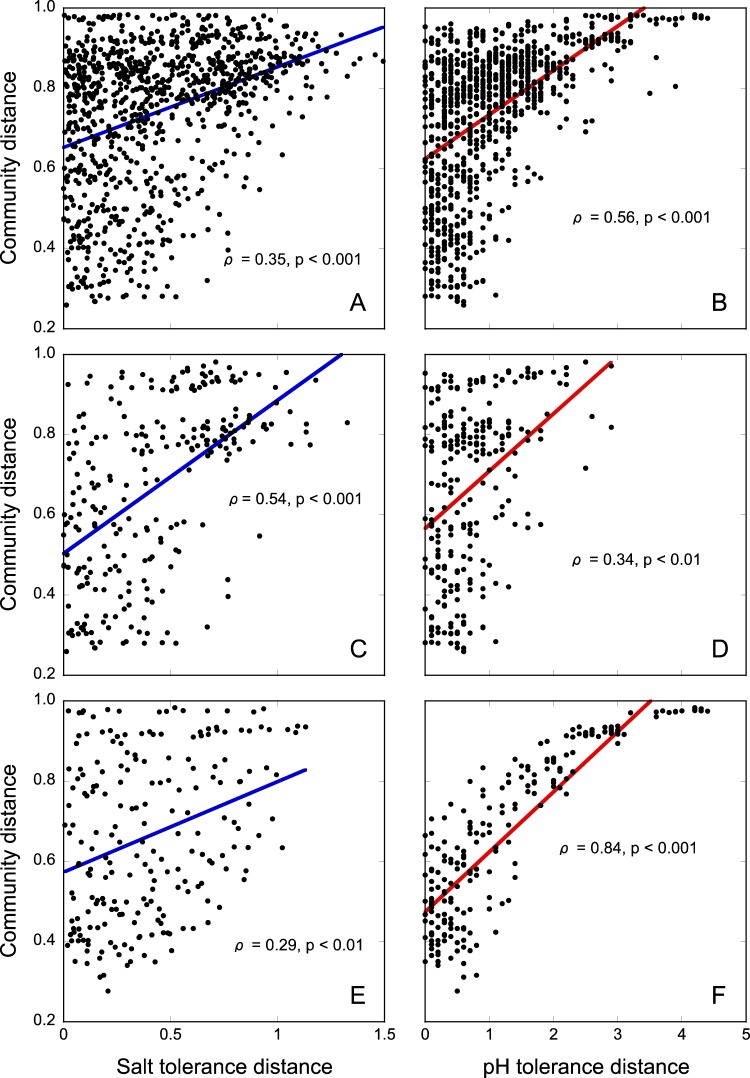
Relationships between pairwise differences in community composition (Bray–Curtis dissimilarities) and community trait distributions (Euclidean distances). The fitted lines show linear regression curves between pairwise community distances and tolerance trait distances. Statistics (*ρ*) were calculated through Mantel tests (Spearman rank correlation). Panels (**a**, **b)** show the correlation between community dissimilarities and salt (**a**) and pH (**b**) tolerance distances for samples from both gradients. Panels (**c**, **d)** include samples from the AG gradient only, while (**e**, **f**) those from the NV gradient only

Community composition changed with increasing salt tolerance along both gradients (Fig. 2, Fig. S5A) and converged between gradients as community salt tolerance increased. Similar patterns were observed when both constrained (Fig. 2) and unconstrained ordination methods (Fig. S5A) were used to visualize patterns in bacterial community composition along the gradients. Both salt and pH tolerance explained significant amounts of community composition, with a higher proportion of variation being related to salt tolerance (Fig. 2). Minor changes in bacterial community composition occurred during the 3-week incubation with plant material used to boost growth rates for tolerance measurements, but the effect of the incubation with plant material was not significant (Fig. S5B). Community composition along both gradients was significantly correlated with salt tolerance (Mantel test; *ρ* = 0.35, *p* *<* 0.001) and pH tolerance (Mantel test; *ρ* = 0.56, *p* < 0.001), with shifts in community composition being better correlated with changes in the trait distribution of pH tolerance than salt tolerance (Fig. 3a, b). Along the AG gradient, community composition was more strongly correlated with salt tolerance (Mantel test; *ρ* = 0.54, *p* < 0.001) than pH tolerance (Mantel test; *ρ* = 0.34, *p* < 0.01) (Fig. 3c, d), whereas along the NV gradient, the correlation between community composition and salt tolerance (Mantel test; *ρ* = 0.29, *p* < 0.01) was weaker than between community composition and pH tolerance (Mantel test; *ρ* = 0.84, *p* < 0.001) (Fig. 3e, f).

Overall, an increase in pH tolerance by 1 pH unit was connected to a shift in community composition of a similar magnitude as the change in community composition associated with an increase of logIC_50_ by 0.5 log (dS m^−^^1^), i.e., a 3.5-fold increase in salt tolerance (Fig. 3a, b). Increases in community salt tolerance were accompanied by larger shifts in community composition along the AG gradient than along the NV gradient. An increase in salt tolerance by 1 log (dS m^−^^1^) (i.e., a 10-fold increase) along the AG gradient (Fig. S7 C) was accompanied by a community shift of the same magnitude as an increase in salt tolerance by ca. 1.7 log (dS m^−^^1^) (i.e. a 50-fold increase) along the NV gradient (Fig. 3e).

In the AG gradient, 46 OTUs with a relative abundance ≥1% were found to be positively correlated with salt tolerance, while 33 OTUs were negatively correlated (Table S1). Fifteen of the 46 positively correlated OTUs belonged to the Gammaproteobacteria and 17 to the phylum Bacteroidetes, making them the taxonomic groups most strongly associated with increased community-level salt tolerance. Among these Gammaproteobacteria were OTUs classified as belonging to the genera *Salinisphaera* and *Alkanibacter* and several OTUs belonging to the families Xanthomonadaceae and Alteromonadaceae (including OTUs classified as *Marinobacter*). The Bacteroidetes associated with high salt tolerance included several Flavobacteriaceae (including OTUs classified as *Gillisia*, *Gramella*, and *Salinimicrobium*), Rhodothermaceae (including *Rubricoccus*), and Flammeovirgaceae, as well as OTUs classified as *Balneola*. In the NV gradient, 19 OTUs were positively correlated with salt tolerance. The class Gammaproteobacteria made up 10 of these 19 OTUs, which were classified as belonging to the families Salinisphaeraceae, Xanthomonadaceae, and Alteromonadacaea. Ten OTUs positively correlated with salinity in the NV gradient were also found to be positively correlated with salt tolerance in the AG gradient (Table S1). Of these 10 OTUs, 9 were only observed in communities with an IC_50_ >1.5 log (dS m^−^^1^).

In the NV gradient, 42 OTUs were positively correlated with pH optimum, while 24 were negatively correlated (Table S2). There was some overlap along the NV gradient between OTUs that responded to salt tolerance and pH optimum: 23 OTUs were positively correlated with pH optimum and negatively correlated with salt tolerance, while 17 OTUs were negatively correlated with pH optimum and positively with salt tolerance (Table S1, S2). OTUs associated with communities with high salt tolerance and low pH optima included mainly members of the phylum Gammaproteobacteria, whereas OTUs associated with communities with low salt tolerance and a high pH optima were more phylogenetically diverse.

Shannon diversity declined along the gradients with increasing salinity and decreasing pH (Fig. S6). The multiple linear regression models for Shannon diversity were significant for both the AG gradient (*F*_(2,20)_ = 14.9, *R*^2^ = 0.56, *p* < 0.001) and the NV gradient (*F*_(2,18)_ = 49.9, *R*^2^ = 0.83, *p* < 0.001). In the AG gradient, both logEC (*p* < 0.001) and pH (*p* < 0.05) significantly predicted Shannon diversity. In the NV gradient, only pH was found to significantly predict Shannon diversity (*p* < 0.001), whereas logEC did not predict a significant proportion of the variation in diversity beyond variation also attributed to pH.

## Discussion

### Trait–environment relationships

The two salinity gradients used in this study cover much of the range of salinities observed across soils globally [39], from non-saline to hypersaline soils, including sites where salt concentrations in the pore water approached saturated conditions. We hypothesized that a community inhabiting a more saline site would have been selected for higher salt tolerance, thereby increasing the amount of salinity required to inhibit growth of that community. We observed substantial and systematic variation in community tolerance to salt along the environmental gradients. In accordance with our hypothesis, community-level salt tolerance increased proportionally to the increase in soil salinity (Fig. 1b). As such, inconsistent links between soil salinity and bacterial salt tolerance that had previously been reported [25, 26] are likely at least partly explained by the small sample sizes and limited ranges in soil salinity considered.

Along the NV gradient, the span in pH ranged from ca. 4.5 to 8.5, encompassing most of the range of pH values that are commonly found in soils around the globe. Soil pH is the factor that was found to be the most strongly correlated with microbial community composition in continental-scale comparisons of soil communities [6, 40]. Similar to the observed increase in community salt tolerance with salinity, we expected community tolerance to pH to shift along the pH gradient. Consistent with our expectation, we found a significant correlation between soil pH and pH_opt_. The observed shifts in community salt tolerance as well as pH tolerance along the gradients (Fig. 1b, d) indicate that both environmental factors had shaped trait distributions and filtered the microbial communities [29, 41]. As such, this approach enabled us to infer a direct impact of the studied environmental factors on the bacterial community, which is not confounded with other factors changing along the gradients, since those would not have resulted in corresponding shifts in tolerance trait distributions [42–44].

### Connecting phenotypic trait distributions and community compositions

One of the main mechanisms through which the trait distribution of tolerance in a community could change includes shifts in community composition, with better adapted species replacing less adapted ones [45, 46]. Changes in bacterial community composition along the gradients that correlate with the salt tolerance of the community are more likely to be a result of a direct influence of salinity, whereas changes in the community with salinity that are not accompanied with shifts in salt tolerance are more likely to be the result of indirect factors covarying with salinity. Compositional changes that accompany shifts in the distribution of traits that are selected upon by the environmental factor in question, in this case salinity, thus provide a far better basis to establish causal links between salinity and community composition than simply testing for correlations between soil salinity and community composition. However, an important caveat to consider is that not all of the observed changes in microbial community composition that were correlated with an increase in salt tolerance would necessarily be due to salinity alone.

As salinity increased along the studied gradients and became a more important constraint on the bacterial community, communities became more similar between samples from different gradients (Fig. 2c). It is likely that only a limited set of bacterial taxa could withstand the considerable stresses imposed on microbial cells in the highly saline soils, which was reflected also in a declining diversity of communities with increasing salinity (Fig. S6). Bacterial communities from different origins that are exposed to the same selection pressure frequently respond in a consistent manner, as has been reported, e.g., in response to heavy metal exposure [47, 48], addition of low molecular weight organic substrate [49], and periodic drying–rewetting events [50], as well as in response to macronutrient addition [51, 52]. As such, this suggests the existence of generalizable and predictable responses to environmental shifts in bacterial communities.

There are conflicting reports on which soil bacterial or archaeal taxa would be expected to increase in response to soil salinity. Previous studies identified a range of different taxa that were positively correlated with salinity [11, 13, 53, 54], which makes it difficult to derive meaningful a priori predictions of bacterial community responses to increasing salinity. In aquatic habitats, high salinity is often associated with a switch toward an archaeal-dominated community [55–57]. In some extremely saline soils, which included more extreme salinities than in this study, high relative abundances of archaea of up to 80% of the community were reported [10, 14]. However, in our study system we saw no change in the abundance of archaeal sequences along the salinity gradients and archaeal sequences never made up ca. >2% of the total number of reads. Canfora et al. [11] found no increase in Proteobacteria but an increasing abundance of Bacteroidetes, while in a study in wetland soils primarily Gamma- and Deltaproteobacteria were associated with higher salinity [54]. Studies in arid and saline soils in Antarctica found the phylum most strongly correlated with salinity to be Firmicutes [13, 53]. We found high community salt tolerance to be positively correlated primarily with OTUs belonging to two high-level phylogenetic groups, namely, the Bacteroidetes and the Gammaproteobacteria, indicating that the trait salt tolerance is represented in these taxonomic groups, with only a few other phyla among the OTUs associated with high salt tolerance. The Gammaproteobacteria that were found to be positively correlated with salinity included phylogenetic groups known to contain halophilic or marine bacteria, e.g., the families Alteromonadaceae [58, 59] and Salinisphaeraceae [60]. Also among the Bacteroidetes that were related to high salt tolerance were taxa belonging to the family Rhodothermaceae, which includes many halophilic bacteria [61], and the Flavobacteriaceae genera *Gillisia*, *Gramella*, and *Salinimicrobium*, which are frequently found in saline environments [62–64]. Taxa from the groups found in high salinity sites of both gradients could be targeted for the use as ‘bioindicator’ taxa for salt-affected communities to assess whether salinity had posed an important selective constraint on the communities. Investigations of these ‘bioindicator’ taxa could be useful in environments where salinity is variable due to irrigation or flooding and current soil salinities do not necessarily reflect the past impact of salinity [65]. In a similar vein, bacterial bioindicator taxa for heavy metal pollution have been put forward by looking for bacterial taxa correlated with high heavy metal pollution [66–68].

### Comparing shifts in community salt and pH tolerance

In our study system, we were able to use the trait–environment relationships established between soil salinity and salt tolerance, as well as between soil pH and pH tolerance to compare the impact of salinity in a terrestrial system to that of pH (factors that are often difficult to disentangle). Salt tolerance accounted for a higher proportion of the total variation in community composition in the total dataset (Fig. 2c). In contrast, along the NV gradient, which was confounded with pH, a larger proportion of the variation in community composition was connected to differences in pH tolerance (Fig. 2b). Overall, bacterial communities whose salt tolerance differed by a factor of 3.5 were as dissimilar in their composition as communities whose pH tolerance differed by 1 pH unit (Fig. 3a, b).

Along the salinity gradients, a larger shift in the community composition per unit change in salt tolerance was observed in the AG gradient (Fig. 3c), which covered a narrower range of salinities than the NV gradient (Fig. 3d). It is possible that, as salinity in the soil solution approached more extreme values in the highly saline sites of the NV gradient, further increases in salinity from already saline conditions resulted in smaller changes in community composition. With salinity becoming the main factor constraining community composition, the pool of species that would have been capable of surviving these extreme conditions and able to outcompete and replace less adapted ones could have decreased [69]. This would imply that increases in salinity in non- or low-saline habitats would result in larger shifts in the bacterial community composition than increases in salinity in already highly saline soils. Similarly, along gradients of heavy metal pollution progressively smaller changes in community composition as levels of heavy metal pollution increased have been reported [67, 70]. In contrast, changes in pH tolerance were connected to shifts in community composition of similar magnitude along both gradients, despite the AG gradient covering a much smaller range of pH values. However, pH values between ca. 5 and 8 are common in soils [6, 40]. Therefore the span of soil pH values covered by the gradients in this study arguably represented less extreme conditions for bacteria than the upper limit of the range of salinities found along the gradients.

## Conclusions

We propose a framework of using bacterial tolerance trait distributions along environmental gradients to identify environmental factors that constitute important filters on bacterial communities and thus infer a causal link . Accompanying these shifts in bacterial tolerance trait distributions along environmental gradients of pH and salinity, we could document large bacterial community differences. As bacterial communities were filtered by salinity, the community compositions grew similar between the two gradients. We identified a high degree of overlap between the two gradients in terms of which taxa were positively correlated with increased salt tolerance, indicative of consistent changes in community composition as bacterial communities became more salt tolerant. These taxa could be used as bioindicators to infer the distribution of salt tolerance in communities derived from environmental samples currently analyzed as part of large-scale survey efforts (including e.g. the Earth Microbiome Project [39]). Identifying such bioindicator taxa makes it possible to use community composition data to predict phenotypic traits (in this case, salt tolerance) of highly diverse soil microbial communities .

## Disclaimer

The submitted material is original research, has not been published previously, and has not been submitted for publication elsewhere while under consideration.

## Electronic supplementary material

Figures S1-S6

Tables S1-S2
