# Supplementary material for: Linking bacterial community composition to soil salinity along environmental gradients
Source: ISME J. 2018 Nov 16;13(3):836–46. doi: 10.1038/s41396-018-0313-8 (PMC6461869; doi:10.1038/s41396-018-0313-8)
Supplement: Supplementary file 1 — Figures S1-S6 [file 41396_2018_313_MOESM1_ESM.docx]

Fig. S1: Satellite image of the sampling sites along the two salinity gradients. The agricultural (AG) gradient was sampled in three transects at the northern shore of the lake in three transects (sampling points 120-127, 130-137 and 140-147). The natural vegetation (NV) gradient was sampled in three transects at the southern shore of the lake (sampling points 150-157, 160-167 and 170-177). Source: Google Earth 7.1.8.3036 (image date 11/26/2015).

Fig.S2: Relationship between electrical conductivity (soil EC) and pH (soil pH) in a 1:5 soil:water mixture along the two salinity gradients. In the AG Gradient soil EC and soil pH were not correlated, whereas along the NV Gradient soil EC and soil pH were found to be significantly negatively correlated (r^2^= 0.50, p<0.001).

Fig. S3: Relationship between pH and organic matter content (as % dry weight (dw) of soil) along the AG and the NV gradient.

Fig. S4: Concentrations of water-soluble cations (Ca^2+^, K^+^, Mg^2+^ and Na^+^) along the salinity gradients


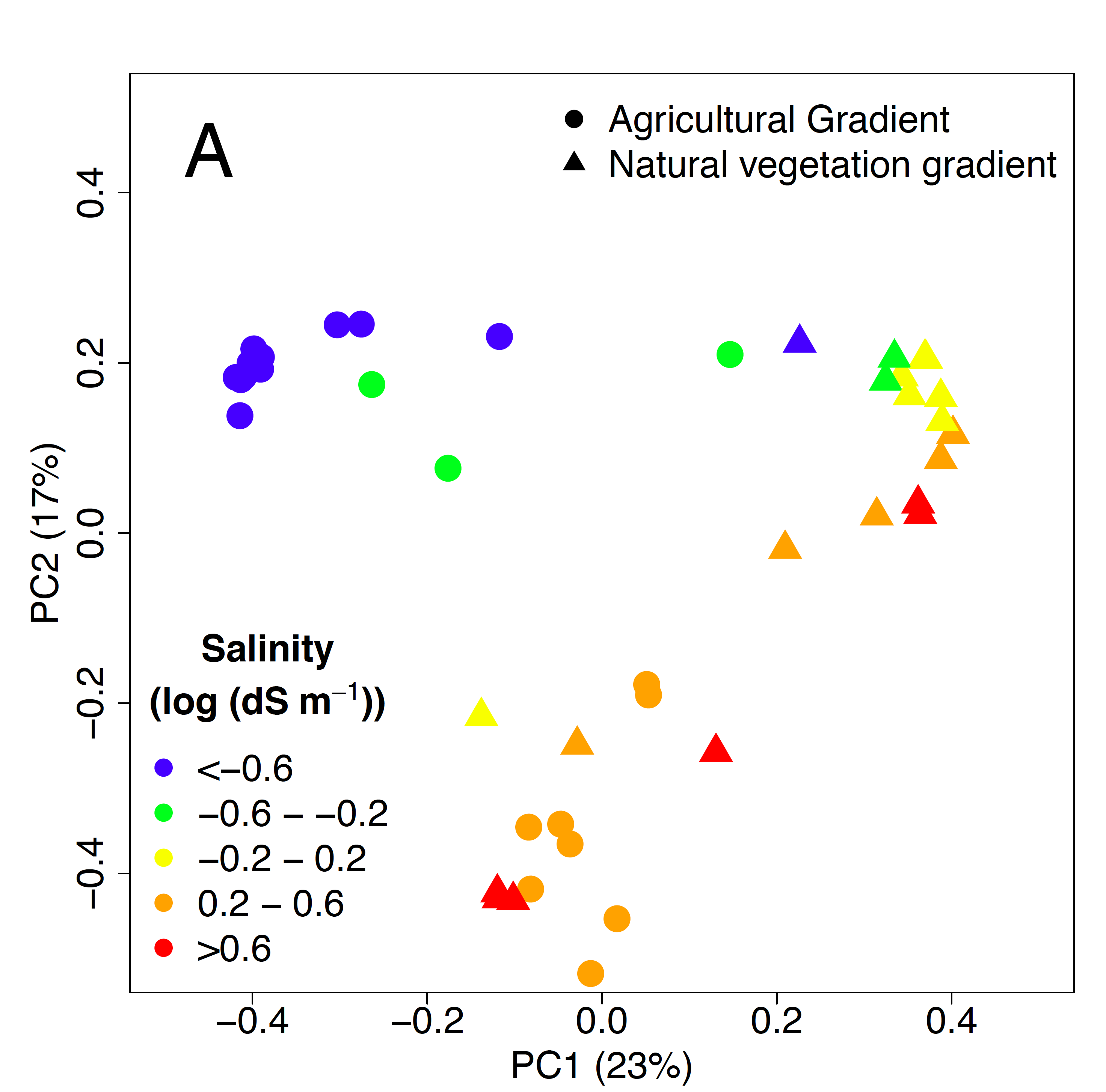

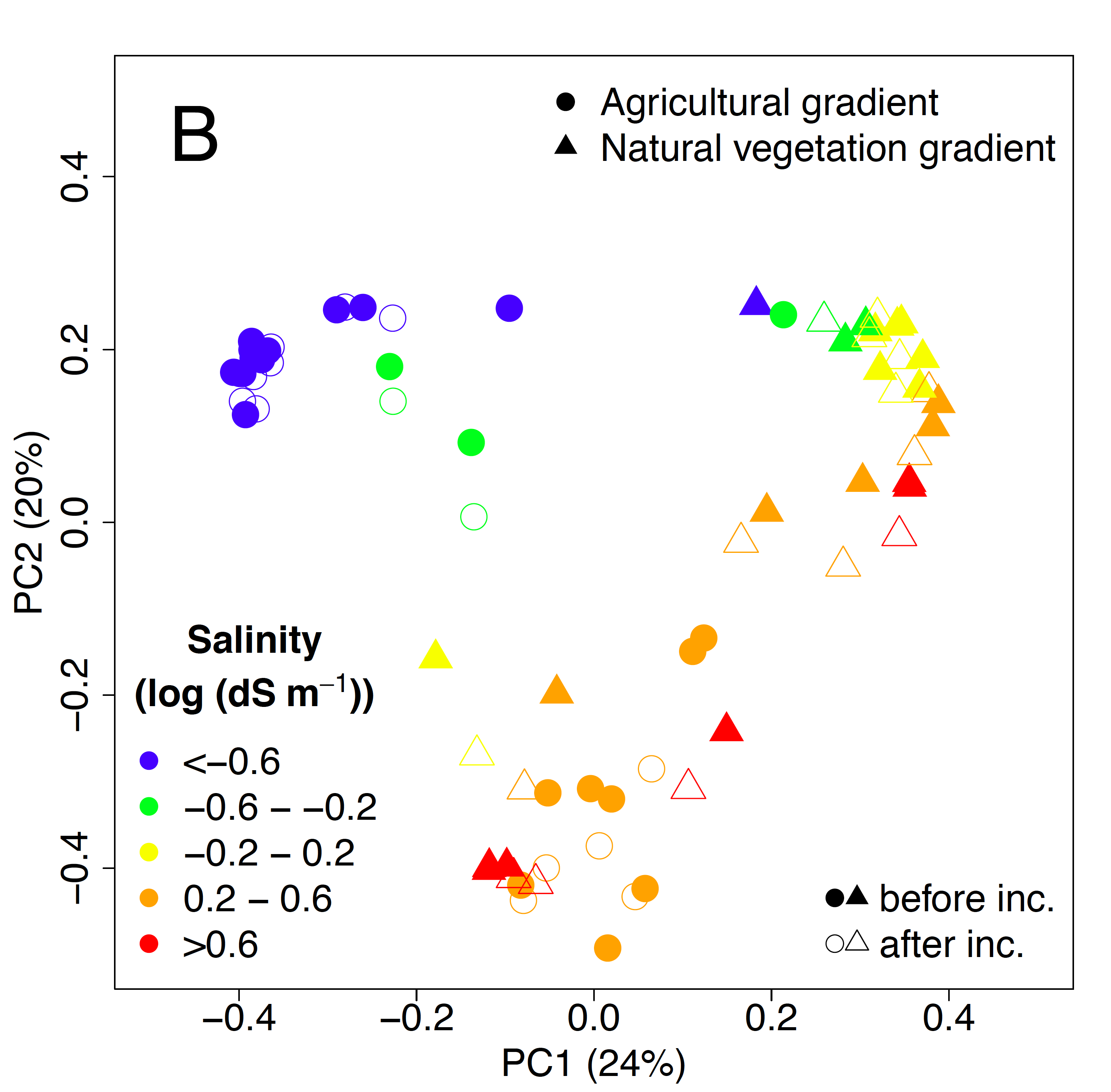


Fig. S5: Principal coordinate analysis of bacterial community composition derived from Bray-Curtis dissimilarities of the community composition of sampling points based on 16S rRNA gene amplicon sequencing. (A) samples from both gradients before incubation with plant material, (B) samples from panel A (full symbols) together with samples that had been incubated with plant material for three weeks (empty symbols). Numbers in parentheses in the axis labels give the percentage of variance accounted for by the principal coordinates.


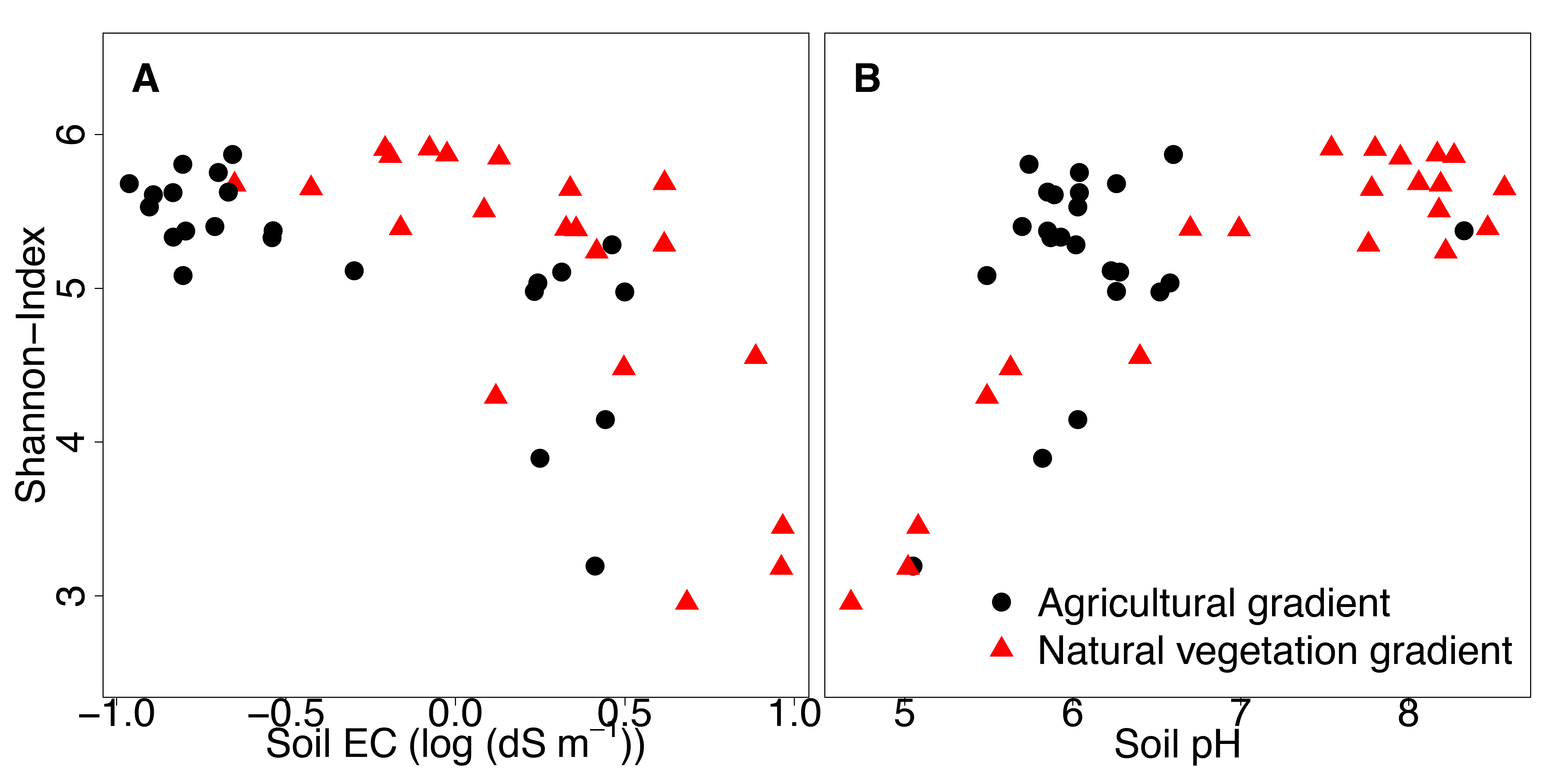


Fig. S6: Shannon diversity against (A) soil electrical conductivity (EC) and (B) soil pH.
