# Supplementary material for: Linking bacterial community composition to soil salinity along environmental gradients
Source: ISME J. 2018 Nov 16;13(3):836–46. doi: 10.1038/s41396-018-0313-8 (PMC6461869; doi:10.1038/s41396-018-0313-8)
Supplement: Supplementary file 2 — Tables S1-S2 [file 41396_2018_313_MOESM2_ESM.docx]

**Supplementary tables**

Table S1: OTUs found to be positively correlated with community salt tolerance along the AG and NV gradient. Selected were OTUs with a maximal abundance of >1% and a Spearman’s correlation coefficient ρ of >0.5. An asterisk next to the OTU-ID denotes that the OTU was found to be positively correlated with salt tolerance in samples from both gradients.

| \| [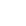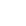](https://en.wikipedia.org/wiki/Rho_(letter)**AG Gradient** \| \| --- \| |  |  |  |
| --- | --- | --- | --- | --- |
| **OTU ID ^1^** | **ρ ^2^** | **Max. abund ^3^.** | **Taxonomy ^4^** |
| OTU_79* | 0.84 | 6.5 | k__Bacteria; p__Proteobacteria; c__Gammaproteobacteria; unclassified; unclassified; unclassified; unclassified |
| OTU_433* | 0.81 | 1.8 | k__Bacteria; p__Proteobacteria; c__Gammaproteobacteria; o__Salinisphaerales; f__Salinisphaeraceae; g__Salinisphaera; s__ |
| OTU_119* | 0.80 | 1.8 | k__Bacteria; p__Gemmatimonadetes; c__Gemm-5; o__; f__; g__; s__ |
| OTU_279 | 0.79 | 7.9 | k__Bacteria; p__Bacteroidetes; c__Flavobacteriia; o__Flavobacteriales; f__Flavobacteriaceae; g__Gillisia; s__ |
| OTU_137 | 0.78 | 1.3 | k__Bacteria; p__Proteobacteria; c__Alphaproteobacteria; o__Rhizobiales; f__Aurantimonadaceae; g__; s__ |
| OTU_336 | 0.78 | 2.8 | k__Bacteria; p__Bacteroidetes; c__[Rhodothermi]; o__[Rhodothermales]; f__[Balneolaceae]; g__KSA1; s__ |
| OTU_396 | 0.77 | 1.1 | k__Bacteria; p__Bacteroidetes; c__[Rhodothermi]; o__[Rhodothermales]; f__Rhodothermaceae; g__Rubricoccus; s__ |
| OTU_407* | 0.77 | 1.5 | k__Bacteria; p__BRC1; c__PRR-11; o__; f__; g__; s__ |
| OTU_944 | 0.76 | 1.5 | k__Bacteria; p__Proteobacteria; c__Gammaproteobacteria; o__Xanthomonadales; f__Xanthomonadaceae; g__; s__ |
| OTU_197 | 0.76 | 1.1 | k__Bacteria; p__Proteobacteria; c__Gammaproteobacteria; o__Xanthomonadales; f__Xanthomonadaceae; unclassified; unclassified |
| OTU_736 | 0.76 | 1.1 | k__Bacteria; p__Bacteroidetes; c__[Rhodothermi]; o__[Rhodothermales]; f__Rhodothermaceae; g__; s__ |
| OTU_225 | 0.75 | 3.5 | k__Bacteria; p__Bacteroidetes; c__Flavobacteriia; o__Flavobacteriales; f__Flavobacteriaceae; unclassified; unclassified |
| OTU_420 | 0.75 | 1.0 | k__Bacteria; p__Proteobacteria; c__Gammaproteobacteria; o__Alteromonadales; f__Alteromonadaceae; g__; s__ |
| OTU_755 | 0.75 | 3.2 | k__Bacteria; p__Bacteroidetes; c__Flavobacteriia; o__Flavobacteriales; f__Flavobacteriaceae; g__Gramella; s__ |
| OTU_13435 | 0.75 | 1.6 | k__Bacteria; p__Proteobacteria; c__Deltaproteobacteria; o__GMD14H09; f__; g__; s__ |
| OTU_1582 | 0.71 | 1.2 | k__Bacteria; p__Bacteroidetes; c__Flavobacteriia; o__Flavobacteriales; f__Flavobacteriaceae; g__Gramella; s__ |
| OTU_468 | 0.71 | 4.2 | k__Bacteria; p__Proteobacteria; c__Gammaproteobacteria; o__Alteromonadales; f__Alteromonadaceae; g__Marinobacter; unclassified |
| OTU_671 | 0.71 | 10.5 | k__Bacteria; p__Bacteroidetes; c__Cytophagia; o__Cytophagales; f__Flammeovirgaceae; g__; s__ |
| OTU_397 | 0.70 | 7.9 | k__Bacteria; p__Proteobacteria; c__Gammaproteobacteria; o__Xanthomonadales; f__Xanthomonadaceae; g__; s__ |
| OTU_154 | 0.70 | 2.3 | k__Bacteria; p__Bacteroidetes; c__Cytophagia; o__Cytophagales; f__Flammeovirgaceae; g__; s__ |
| OTU_523 | 0.70 | 1.4 | k__Bacteria; p__Proteobacteria; c__Gammaproteobacteria; o__Xanthomonadales; f__Xanthomonadaceae; g__; s__ |
| OTU_124* | 0.69 | 3.6 | k__Bacteria; p__Bacteroidetes; c__[Rhodothermi]; o__[Rhodothermales]; f__[Balneolaceae]; g__KSA1; s__ |
| OTU_467 | 0.65 | 3.4 | k__Bacteria; p__Proteobacteria; c__Gammaproteobacteria; unclassified; unclassified; unclassified; unclassified |
| OTU_103 | 0.65 | 14.8 | k__Bacteria; p__Bacteroidetes; c__[Rhodothermi]; o__[Rhodothermales]; f__[Balneolaceae]; g__KSA1; s__ |
| OTU_429 | 0.61 | 2.8 | k__Bacteria; p__Bacteroidetes; c__Flavobacteriia; o__Flavobacteriales; f__Cryomorphaceae; g__Owenweeksia; s__ |
| OTU_532 | 0.61 | 1.4 | k__Bacteria; p__Proteobacteria; c__Gammaproteobacteria; o__Alteromonadales; f__Alteromonadaceae; g__; s__ |
| OTU_9051 | 0.60 | 1.5 | k__Bacteria; p__Proteobacteria; c__Gammaproteobacteria; unclassified; unclassified; unclassified; unclassified |
| OTU_676 | 0.60 | 1.9 | k__Bacteria; p__Proteobacteria; c__Alphaproteobacteria; o__Rhizobiales; f__Hyphomicrobiaceae; g__Devosia; s__ |
| OTU_339 | 0.60 | 2.6 | k__Bacteria; p__Gemmatimonadetes; c__Gemm-3; o__; f__; g__; s__ |
| OTU_111 | 0.60 | 1.5 | k__Bacteria; p__Bacteroidetes; c__[Rhodothermi]; o__[Rhodothermales]; f__[Balneolaceae]; unclassified; unclassified |
| OTU_267* | 0.59 | 3.7 | k__Bacteria; p__Proteobacteria; c__Gammaproteobacteria; o__Salinisphaerales; f__Salinisphaeraceae; g__; s__ |
| OTU_17369* | 0.59 | 6.2 | k__Bacteria; p__Proteobacteria; c__Alphaproteobacteria; o__Sphingomonadales; f__Sphingomonadaceae; g__; s__ |
| OTU_1052 | 0.59 | 21.8 | k__Bacteria; p__Proteobacteria; c__Gammaproteobacteria; o__Xanthomonadales; f__Sinobacteraceae; g__Alkanibacter; s__difficilis |
| OTU_327 | 0.58 | 1.0 | k__Bacteria; p__Proteobacteria; c__Alphaproteobacteria; o__Sphingomonadales; f__Sphingomonadaceae; g__Kaistobacter; s__ |
| OTU_5388 | 0.58 | 7.3 | k__Bacteria; p__Bacteroidetes; c__Flavobacteriia; o__Flavobacteriales; f__Cryomorphaceae; g__; s__ |
| OTU_441 | 0.57 | 2.7 | k__Bacteria; p__Proteobacteria; c__Deltaproteobacteria; o__GMD14H09; f__; g__; s__ |
| OTU_675 | 0.56 | 6.0 | k__Bacteria; p__Proteobacteria; c__Gammaproteobacteria; o__Oceanospirillales; f__Alcanivoracaceae; g__Alcanivorax; unclassified |
| OTU_768 | 0.55 | 1.5 | k__Bacteria; p__Bacteroidetes; c__[Saprospirae]; o__[Saprospirales]; f__Chitinophagaceae; g__; s__ |
| OTU_332 | 0.55 | 2.1 | k__Bacteria; p__Bacteroidetes; c__Flavobacteriia; o__Flavobacteriales; f__Flavobacteriaceae; g__Salinimicrobium; s__ |
| OTU_800 | 0.55 | 1.6 | k__Bacteria; p__Proteobacteria; c__Alphaproteobacteria; o__Rhizobiales; f__Hyphomicrobiaceae; g__; s__ |
| OTU_50* | 0.55 | 1.1 | k__Bacteria; p__Firmicutes; c__Bacilli; o__Bacillales; f__Sporolactobacillaceae; g__; s__ |
| OTU_31* | 0.54 | 2.6 | k__Bacteria; p__Chlorobi; c__Ignavibacteria; o__Ignavibacteriales; f__Ignavibacteriaceae; g__; s__ |
| OTU_253* | 0.53 | 3.5 | k__Bacteria; p__Bacteroidetes; c__[Rhodothermi]; o__[Rhodothermales]; f__[Balneolaceae]; g__Balneola; s__ |
| OTU_167 | 0.51 | 1.4 | k__Bacteria; p__Proteobacteria; c__Gammaproteobacteria; o__Xanthomonadales; f__Xanthomonadaceae; unclassified; unclassified |
| OTU_902 | 0.51 | 1.2 | k__Bacteria; p__TM6; c__SJA-4; o__; f__; g__; s__ |
| OTU_1290 | 0.51 | 1.4 | k__Archaea; p__Euryarchaeota; c__Halobacteria; o__Halobacteriales; f__Halobacteriaceae; g__Halococcus; s__ |
| **[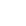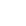](https://en.wikipedia.org/wiki/Rho_(letter)**   \| **NV Gradient** \| \| --- \| |  |  |  |
| **OTU ID** | **ρ** | **Max. abundance** | **Taxonomy** |
| OTU_79* | 0.69 | 41.2 | k__Bacteria; p__Proteobacteria; c__Gammaproteobacteria; unclassified; unclassified; unclassified; unclassified |
| OTU_50* | 0.68 | 9.9 | k__Bacteria; p__Proteobacteria; c__Gammaproteobacteria; o__Salinisphaerales; f__Salinisphaeraceae; g__Salinisphaera; s__ |
| OTU_267* | 0.66 | 6.1 | k__Bacteria; p__Proteobacteria; c__Gammaproteobacteria; unclassified; unclassified; unclassified; unclassified |
| OTU_10249 | 0.65 | 3.3 | k__Bacteria; p__Proteobacteria; c__Gammaproteobacteria; o__Xanthomonadales; f__Xanthomonadaceae; unclassified; unclassified |
| OTU_119* | 0.64 | 5.0 | k__Bacteria; p__Bacteroidetes; c__[Rhodothermi]; o__[Rhodothermales]; f__[Balneolaceae]; g__KSA1; s__ |
| OTU_288 | 0.61 | 1.8 | k__Bacteria; p__Proteobacteria; c__Alphaproteobacteria; o__Sphingomonadales; f__Erythrobacteraceae; g__; s__ |
| OTU_639 | 0.58 | 2.9 | k__Bacteria; p__Chloroflexi; c__Ktedonobacteria; o__B12-WMSP1; f__; g__; s__ |
| OTU_5818 | 0.58 | 1.1 | k__Bacteria; p__Actinobacteria; c__Actinobacteria; o__Actinomycetales; unclassified; unclassified; unclassified |
| OTU_8324 | 0.57 | 2.9 | k__Bacteria; p__Proteobacteria; c__Gammaproteobacteria; o__Xanthomonadales; f__Xanthomonadaceae; g__Luteibacter; s__rhizovicinus |
| OTU_17369* | 0.57 | 3.7 | k__Bacteria; p__Proteobacteria; c__Gammaproteobacteria; o__Xanthomonadales; f__Xanthomonadaceae; unclassified; unclassified |
| OTU_4189 | 0.57 | 5.0 | k__Bacteria; p__Proteobacteria; c__Gammaproteobacteria; unclassified; unclassified; unclassified; unclassified |
| OTU_668 | 0.55 | 2.2 | k__Bacteria; p__Bacteroidetes; c__[Rhodothermi]; o__[Rhodothermales]; f__[Balneolaceae]; unclassified; unclassified |
| OTU_124* | 0.54 | 11.0 | k__Bacteria; p__Proteobacteria; c__Gammaproteobacteria; o__Xanthomonadales; f__Xanthomonadaceae; g__; s__ |
| OTU_433* | 0.53 | 1.7 | k__Bacteria; p__Gemmatimonadetes; c__Gemm-5; o__; f__; g__; s__ |
| OTU_407* | 0.53 | 2.2 | k__Bacteria; p__Proteobacteria; c__Gammaproteobacteria; o__Xanthomonadales; f__Xanthomonadaceae; unclassified; unclassified |
| OTU_823 | 0.52 | 1.4 | k__Bacteria; p__Planctomycetes; c__Planctomycetia; o__Gemmatales; f__Isosphaeraceae; g__; s__ |
| OTU_319 | 0.52 | 5.5 | k__Bacteria; p__Proteobacteria; c__Gammaproteobacteria; o__Alteromonadales; f__Alteromonadaceae; g__Marinobacter; unclassified |
| OTU_253* | 0.51 | 11.9 | k__Bacteria; p__Bacteroidetes; c__[Rhodothermi]; o__[Rhodothermales]; f__[Balneolaceae]; unclassified; unclassified |
| OTU_31* | 0.51 | 1.1 | k__Bacteria; p__Firmicutes; c__Bacilli; o__Bacillales; f__Sporolactobacillaceae; g__; s__ |

^1^ Number assigned to each operational taxonomic unit (OTU)

^2^ Spearman rank correlation coefficient ρ

^3^ Maximum abundance of each OTU in the gradient in % of reads

^4^ Taxonomy assigned to OTU: k= kingdom, p = phylum, c= class, o=order, f=family, g=genus, s=species

Table S2: OTUs found to be positively and negatively correlated with pH_opt_ of bacterial communities along the NV gradient. Selected were OTUs with a maximal abundance of >1% and a Spearman’s correlation coefficient ρ of >0.5. OTUs with a positive correlation increased in abundance with a high pH_opt_ of the community, whereas OTUs with a negative correlation increased in abundance with a low pH_opt_.

| \| [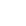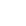](https://en.wikipedia.org/wiki/Rho_(letter)**Positive correlation** \| \| --- \| |  |  |  |
| --- | --- | --- | --- | --- |
| **OTU ID ^1^** | **ρ ^2^** | **Max. abund ^3^.** | **Taxonomy ^4^** |
| OTU_501 | 0.83 | 5.4 | k__Bacteria; p__Proteobacteria; c__Deltaproteobacteria; o__Myxococcales; f__Haliangiaceae; g__Haliangium; s__ |
| OTU_386 | 0.81 | 3.7 | k__Bacteria; p__Gemmatimonadetes; c__Gemm-5; o__; f__; g__; s__ |
| OTU_514 | 0.81 | 2.0 | k__Bacteria; p__Bacteroidetes; c__Sphingobacteriia; o__Sphingobacteriales; f__; g__; s__ |
| OTU_451 | 0.77 | 1.5 | k__Archaea; p__Crenarchaeota; c__Thaumarchaeota; o__Nitrososphaerales; f__Nitrososphaeraceae; g__Candidatus Nitrososphaera; s__gargensis |
| OTU_590 | 0.76 | 1.5 | k__Bacteria; p__Bacteroidetes; c__Cytophagia; o__Cytophagales; f__Cytophagaceae; g__; s__ |
| OTU_240 | 0.75 | 2.3 | k__Bacteria; p__Proteobacteria; c__Deltaproteobacteria; o__Syntrophobacterales; f__Syntrophobacteraceae; g__; s__ |
| OTU_1161 | 0.75 | 1.0 | k__Bacteria; p__Planctomycetes; c__Phycisphaerae; o__WD2101; f__; g__; s__ |
| OTU_372 | 0.75 | 1.4 | k__Archaea; p__Crenarchaeota; c__Thaumarchaeota; o__Nitrososphaerales; f__Nitrososphaeraceae; g__Candidatus Nitrososphaera; s__ |
| OTU_817 | 0.75 | 1.2 | k__Bacteria; p__Gemmatimonadetes; c__Gemm-5; o__; f__; g__; s__ |
| OTU_540 | 0.74 | 1.5 | k__Bacteria; p__Proteobacteria; c__Gammaproteobacteria; o__Xanthomonadales; f__Sinobacteraceae; g__Steroidobacter; s__ |
| OTU_282 | 0.73 | 2.0 | k__Bacteria; p__Proteobacteria; c__Gammaproteobacteria; o__Xanthomonadales; f__Xanthomonadaceae; g__; s__ |
| OTU_292 | 0.72 | 2.7 | k__Bacteria; p__Gemmatimonadetes; c__Gemm-1; o__; f__; g__; s__ |
| OTU_521 | 0.71 | 2.4 | k__Bacteria; p__Verrucomicrobia; c__[Pedosphaerae]; o__[Pedosphaerales]; f__; g__; s__ |
| OTU_370 | 0.70 | 1.7 | k__Bacteria; p__Gemmatimonadetes; c__Gemm-3; o__; f__; g__; s__ |
| OTU_975 | 0.70 | 1.5 | k__Bacteria; p__Verrucomicrobia; c__[Pedosphaerae]; o__[Pedosphaerales]; f__Ellin517; g__; s__ |
| OTU_477 | 0.70 | 1.6 | k__Bacteria; p__Bacteroidetes; c__Cytophagia; o__Cytophagales; f__Cytophagaceae; g__; s__ |
| OTU_574 | 0.69 | 1.1 | k__Bacteria; p__Gemmatimonadetes; c__Gemm-5; o__; f__; g__; s__ |
| OTU_863 | 0.69 | 1.4 | k__Bacteria; p__Proteobacteria; c__Alphaproteobacteria; unclassified; unclassified; unclassified; unclassified |
| OTU_1098 | 0.67 | 1.6 | k__Bacteria; p__Planctomycetes; c__Planctomycetia; o__Pirellulales; f__Pirellulaceae; g__; s__ |
| OTU_423 | 0.67 | 2.5 | k__Bacteria; p__Proteobacteria; c__Gammaproteobacteria; o__Thiotrichales; f__Piscirickettsiaceae; g__; s__ |
| OTU_384 | 0.66 | 1.4 | k__Bacteria; p__Actinobacteria; c__Acidimicrobiia; o__Acidimicrobiales; f__; g__; s__ |
| OTU_10225 | 0.66 | 1.0 | k__Bacteria; p__Proteobacteria; c__Gammaproteobacteria; o__Thiotrichales; f__Piscirickettsiaceae; g__; s__ |
| OTU_534 | 0.65 | 1.9 | k__Bacteria; p__Proteobacteria; c__Alphaproteobacteria; o__Sphingomonadales; f__Sphingomonadaceae; g__Kaistobacter; s__ |
| OTU_199 | 0.64 | 8.6 | k__Bacteria; p__Proteobacteria; c__Gammaproteobacteria; o__Chromatiales; f__; g__; s__ |
| OTU_246 | 0.63 | 5.0 | k__Bacteria; p__Bacteroidetes; c__Cytophagia; o__Cytophagales; f__Flammeovirgaceae; g__; s__ |
| OTU_710 | 0.61 | 1.4 | k__Bacteria; p__Proteobacteria; c__Alphaproteobacteria; o__Rhizobiales; f__Rhodobiaceae; g__Afifella; s__ |
| OTU_518 | 0.61 | 1.7 | k__Bacteria; p__Planctomycetes; c__Planctomycetia; o__Planctomycetales; f__Planctomycetaceae; g__Planctomyces; s__ |
| OTU_1333 | 0.61 | 1.3 | k__Bacteria; p__Gemmatimonadetes; c__Gemm-5; o__; f__; g__; s__ |
| OTU_616 | 0.61 | 1.1 | k__Bacteria; p__Acidobacteria; c__Acidobacteria-6; o__iii1-15; f__; g__; s__ |
| OTU_732 | 0.60 | 1.1 | k__Bacteria; p__Bacteroidetes; c__Cytophagia; o__Cytophagales; f__Cytophagaceae; g__; s__ |
| OTU_276 | 0.59 | 2.7 | k__Bacteria; p__Actinobacteria; c__Rubrobacteria; o__Rubrobacterales; f__Rubrobacteraceae; g__Rubrobacter; s__ |
| OTU_471 | 0.58 | 1.7 | k__Bacteria; p__Proteobacteria; c__Gammaproteobacteria; o__Xanthomonadales; f__Xanthomonadaceae; g__; s__ |
| OTU_381 | 0.57 | 3.3 | k__Bacteria; p__Bacteroidetes; c__[Rhodothermi]; o__[Rhodothermales]; f__Rhodothermaceae; g__; s__ |
| OTU_603 | 0.57 | 2.6 | k__Bacteria; p__Acidobacteria; c__[Chloracidobacteria]; o__RB41; f__; g__; s__ |
| OTU_472 | 0.57 | 1.1 | k__Bacteria; p__Proteobacteria; c__Deltaproteobacteria; o__Syntrophobacterales; f__Syntrophobacteraceae; g__; s__ |
| OTU_1431 | 0.57 | 1.3 | k__Bacteria; p__Proteobacteria; c__Gammaproteobacteria; o__Xanthomonadales; f__Xanthomonadaceae; g__Lysobacter; s__ |
| OTU_263 | 0.56 | 2.7 | k__Bacteria; p__Actinobacteria; c__Rubrobacteria; o__Rubrobacterales; f__Rubrobacteraceae; g__Rubrobacter; s__ |
| OTU_775 | 0.55 | 2.4 | k__Bacteria; p__Proteobacteria; c__Alphaproteobacteria; o__Rhodospirillales; unclassified; unclassified; unclassified |
| OTU_845 | 0.54 | 1.1 | k__Bacteria; p__Proteobacteria; c__Deltaproteobacteria; o__Myxococcales; f__Haliangiaceae; g__; s__ |
| OTU_9871 | 0.52 | 1.1 | k__Bacteria; p__Proteobacteria; c__Alphaproteobacteria; o__Sphingomonadales; f__Sphingomonadaceae; g__Kaistobacter; s__ |
| OTU_821 | 0.52 | 1.2 | k__Bacteria; p__Verrucomicrobia; c__[Spartobacteria]; o__[Chthoniobacterales]; f__[Chthoniobacteraceae]; g__DA101; s__ |
| OTU_134 | 0.50 | 1.1 | k__Bacteria; p__Proteobacteria; c__Alphaproteobacteria; o__Sphingomonadales; f__Sphingomonadaceae; g__Kaistobacter; s__ |
| **[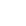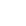](https://en.wikipedia.org/wiki/Rho_(letter)**   \| **Negative correlation** \| \| --- \| |  |  |  |
| **OTU ID** | **ρ** | **Max. abundance** | **Taxonomy** |
| OTU_79 | -0.87 | 41 | k__Bacteria; p__Proteobacteria; c__Gammaproteobacteria; unclassified; unclassified; unclassified; unclassified |
| OTU_119 | -0.80 | 5.0 | k__Bacteria; p__Bacteroidetes; c__[Rhodothermi]; o__[Rhodothermales]; f__[Balneolaceae]; g__KSA1; s__ |
| OTU_267 | -0.80 | 6.1 | k__Bacteria; p__Proteobacteria; c__Gammaproteobacteria; unclassified; unclassified; unclassified; unclassified |
| OTU_10249 | -0.79 | 3.3 | k__Bacteria; p__Proteobacteria; c__Gammaproteobacteria; o__Xanthomonadales; f__Xanthomonadaceae; unclassified; unclassified |
| OTU_31 | -0.76 | 1.1 | k__Bacteria; p__Firmicutes; c__Bacilli; o__Bacillales; f__Sporolactobacillaceae; g__; s__ |
| OTU_823 | -0.74 | 1.4 | k__Bacteria; p__Planctomycetes; c__Planctomycetia; o__Gemmatales; f__Isosphaeraceae; g__; s__ |
| OTU_8324 | -0.74 | 2.9 | k__Bacteria; p__Proteobacteria; c__Gammaproteobacteria; o__Xanthomonadales; f__Xanthomonadaceae; g__Luteibacter; s__rhizovicinus |
| OTU_17369 | -0.74 | 3.7 | k__Bacteria; p__Proteobacteria; c__Gammaproteobacteria; o__Xanthomonadales; f__Xanthomonadaceae; unclassified; unclassified |
| OTU_50 | -0.73 | 9.9 | k__Bacteria; p__Proteobacteria; c__Gammaproteobacteria; o__Salinisphaerales; f__Salinisphaeraceae; g__Salinisphaera; s__ |
| OTU_17 | -0.71 | 1.3 | k__Bacteria; p__Proteobacteria; c__Gammaproteobacteria; o__Xanthomonadales; f__Xanthomonadaceae; g__Dokdonella; s__ |
| OTU_433 | -0.69 | 1.7 | k__Bacteria; p__Gemmatimonadetes; c__Gemm-5; o__; f__; g__; s__ |
| OTU_639 | -0.69 | 2.9 | k__Bacteria; p__Chloroflexi; c__Ktedonobacteria; o__B12-WMSP1; f__; g__; s__ |
| OTU_5818 | -0.69 | 1.1 | k__Bacteria; p__Actinobacteria; c__Actinobacteria; o__Actinomycetales; unclassified; unclassified; unclassified |
| OTU_319 | -0.67 | 5.5 | k__Bacteria; p__Proteobacteria; c__Gammaproteobacteria; o__Alteromonadales; f__Alteromonadaceae; g__Marinobacter; unclassified |
| OTU_91 | -0.67 | 14.7 | k__Bacteria; p__Bacteroidetes; c__[Saprospirae]; o__[Saprospirales]; f__Chitinophagaceae; g__; s__ |
| OTU_124 | -0.65 | 11.0 | k__Bacteria; p__Proteobacteria; c__Gammaproteobacteria; o__Xanthomonadales; f__Xanthomonadaceae; g__; s__ |
| OTU_167 | -0.64 | 8.1 | k__Bacteria; p__Proteobacteria; c__Alphaproteobacteria; o__Sphingomonadales; f__Sphingomonadaceae; g__; s__ |
| OTU_668 | -0.61 | 2.2 | k__Bacteria; p__Bacteroidetes; c__[Rhodothermi]; o__[Rhodothermales]; f__[Balneolaceae]; unclassified; unclassified |
| OTU_154 | -0.61 | 2.4 | k__Bacteria; p__Proteobacteria; c__Gammaproteobacteria; o__Oceanospirillales; f__Alcanivoracaceae; g__Alcanivorax; unclassified |
| OTU_4189 | -0.60 | 5.0 | k__Bacteria; p__Proteobacteria; c__Gammaproteobacteria; unclassified; unclassified; unclassified; unclassified |
| OTU_279 | -0.60 | 1.1 | k__Bacteria; p__Bacteroidetes; c__[Rhodothermi]; o__[Rhodothermales]; f__[Balneolaceae]; g__KSA1; s__ |
| OTU_5881 | -0.58 | 1.5 | k__Bacteria; p__Actinobacteria; c__Actinobacteria; o__Actinomycetales; f__Frankiaceae; g__; s__ |
| OTU_407 | -0.54 | 2.2 | k__Bacteria; p__Proteobacteria; c__Gammaproteobacteria; o__Xanthomonadales; f__Xanthomonadaceae; unclassified; unclassified |
| OTU_137 | -0.53 | 1.4 | k__Bacteria; p__Proteobacteria; c__Gammaproteobacteria; o__Alteromonadales; f__Alteromonadaceae; g__Marinobacter; unclassified |

^1^ Number assigned to each operational taxonomic unit (OTU)

^2^ Spearman rank correlation coefficient ρ between OTU abundance and pH_opt_

^3^ Maximum abundance of each OTU in the gradient in % of reads

^4^ Taxonomy assigned to OTU: k= kingdom, p = phylum, c= class, o=order, f=family, g=genus, s=species
